# Air temperature drives the evolution of mid-infrared optical properties of butterfly wings

**DOI:** 10.1038/s41598-021-02810-1

**Published:** 2021-12-17

**Authors:** Anirudh Krishna, Xiao Nie, Adriana D. Briscoe, Jaeho Lee

**Affiliations:** 1 Intel Corporation, Hillsboro, OR 97124 USA; 2 Department of Mechanical and Aerospace Engineering, University of California, Irvine, CA 92697 USA; 3 Department of Ecology and Evolutionary Biology, University of California, Irvine, CA 92697 USA

**Keywords:** Evolution, Ecology, Environmental sciences, Engineering, Optics and photonics

## Abstract

This study uncovers a correlation between the mid-infrared emissivity of butterfly wings and the average air temperature of their habitats across the world. Butterflies from cooler climates have a lower mid-infrared emissivity, which limits heat losses to surroundings, and butterflies from warmer climates have a higher mid-infrared emissivity, which enhances radiative cooling. The mid-infrared emissivity showed no correlation with other investigated climatic factors. Phylogenetic independent contrasts analysis indicates the microstructures of butterfly wings may have evolved in part to regulate mid-infrared emissivity as an adaptation to climate, rather than as phylogenetic inertia. Our findings offer new insights into the role of microstructures in thermoregulation and suggest both evolutionary and physical constraints to butterflies’ abilities to adapt to climate change.

## Introduction

Scientists and engineers have long been fascinated by the dazzling array of optical properties present in nature^1–13^. While coloration is a tangible reminder of the wonders of microstructures in animals, microstructures may also play a significant role in thermoregulation. Many animals take advantage of structural thermoregulation to thrive in diverse habitat climates around the world. Saharan silver ants, for instance, use surface microstructures to stay cool under desert sunlight by simultaneously reflecting solar heat and maximizing heat emission from their bodies in the mid-infrared (mid-IR) wavelengths^14^. Meanwhile, polar bears stay warm in the cold Arctic due to their dense fur, which is not just a warm blanket but is also a radiative layer that scatters IR radiation and minimizes heat loss^15^. As in other animals, we observe similar optical and thermal phenomena in butterflies, where wing microstructures affect not only coloration^16–18^ but also assist in the thermoregulation of these insects^19,20^. The variation in optical properties that facilitates thermoregulation in butterflies is the result of a combination of pigmentation and chitin-based photonic structures^17,21–25^.

The focus of the majority of optical and thermal studies on butterflies has been on visible to near-IR wavelengths (0.4–2.5 µm wavelengths) where absorption of heat from the incident sunlight takes place. Munro et al. ^26^ examined the correlation of the visible to near-IR optical properties of butterfly wings to the butterfliesʼ habitat temperature and precipitation, and showed that near-IR wing reflectance, rather than UV–Vis reflectance, is correlated with temperature. Nonetheless, our understanding of the optical properties of butterfly wing microstructures in mid-IR wavelengths beyond the visible to near-IR remains limited.

While the visible to near-IR wavelengths are important for heat gain via absorption of incident solar irradiation, the mid-IR wavelengths of 7.5–14 µm are critical for heat loss. The mid-IR wavelengths correspond to the wavelength range where thermal emission takes place as governed by Planck’s law^27–29^. Moreover, this wavelength region also hosts the window of atmospheric transmission^30^, which can lead to substantial radiative cooling in the ambient environment to outer space at 3 K (–270 °C)^31,32^. Importantly, this part of the spectrum (mid-IR) has not yet been systematically examined in butterflies although the effect of mid-IR radiative cooling has been examined in engineering systems^31,33,34^ and in other biological taxa^14,35,36^ in recent years.

Butterflies, being cold-blooded, regulate their body temperature using strategies ranging from behavioral adaptations to reliance on habitat and environmental conditions^22,37–40^. Thermoregulation is crucial in butterflies because they must maintain a body temperature of 20–50 °C in order to survive, regardless of where their habitat is located^41–43^. To attain a stable body temperature in such geographically and climatically diverse locations as the tropics and the Arctic, there is a need for passive thermoregulation in butterfly wings. Specifically, butterflies heat up their wings (and hence, bodies) by absorption of solar heat in the ultraviolet (UV) to near-IR wavelengths. Simultaneously, in order to avoid overheating, they cool down by utilizing re-emission of heat to outer space from the mid-IR wavelengths^19,39,44^. To effectively regulate their wing (and body) temperatures, butterflies utilize photonic structures^2,21,24,45,46^ to not only control visible and near-IR properties but also invisible mid-IR properties^16,22,47,48^ for thermoregulation. These microstructures may consequently be an adaptation of the different butterflies to their varying climates. Consequently, in order to understand how the butterflies thrive in diverse habitats, it is vital to understand the correlation between the microstructure-dependent mid-IR optical properties of the butterfly wings and their respective habitat climates.

Emerging studies have aimed to address our lack of understanding of the mid-IR optical properties of butterfly wings^19,20,44^ with increasing evidence being presented for the role played by wing microstructures in butterfly thermoregulation. Not only do the microstructures aid in the re-emission of heat from butterfly wings, but a matrix of living cells on butterfly wings coordinates with the microstructures to maintain a viable temperature for the butterfly^20^. Meanwhile, other findings have shown that different butterfly species have unique wing microstructures that are critical to how the mid-IR optical properties vary across butterflies from diverse climates^19^. These works have led to a greater knowledge of thermoregulation mechanisms in butterflies. However, there remains much to be learned about the adaptation of butterflies across vastly differing habitats to achieve thermoregulation via wing microstructures.

Differing environmental and climate conditions across habitats impose varying radiative demands on butterflies. Here our computational results for the optical properties of butterfly wings demonstrate the potential use of wing microstructures to regulate wing temperatures, enabling enhanced thermal performance and allowing for the survival of butterflies across the globe. The study of how nature adapts to extremes in climate is of vital importance to our own survival in such habitats. Recent advancements have been made in the fields of engineered materials and systems that make use of bio-inspired structures to achieve thermal control in various ambient climatic conditions^49–52^. By analyzing the thermoregulation strategies of butterflies around the world we might thus find better engineered solutions for our own lives.

## Results

### Structural control of spectral optical properties of butterfly wings

We investigated microstructure-driven optical properties by performing computations based on rigorous coupled-wave analysis (RCWA) and finite-difference time-domain (FDTD) methods. Both methods of analysis solve for Maxwell’s equations, with choices being dependent upon time and computational complexity requirements. We primarily used RCWA for parametric analysis and FDTD for validation^32,49,52^.

**Figure 1:**
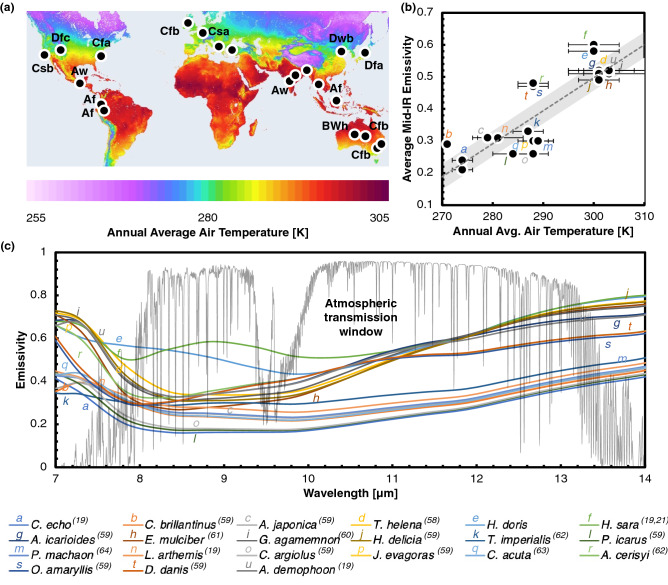
Butterfly habitat annual average air temperature and butterfly wing emissivity (**a**) Geographical mapping of the sampled butterfly species from around the world, generated using open source QGIS software v.3.20.3 (www.qgis.org). The coloration of the map indicates the annual average ambient air temperature^90^. The text adjacent to the data points indicates the habitat-related Köppen-Geiger climate classification^72^. Here, A is a tropical climate, B is arid, C is temperate, and D is continental, with W being a desert, w indicating a dry winter, s a dry summer, f meaning no dry season, a, b, and c pointing to hot, warm, and cold summers, and h indicating an overall hot climate year-round. (**b**) Correlation of average mid-IR emissivity of the wings of butterflies with annual average habitat air temperature. The computed mid-IR emissivity was averaged over 7.5–14 µm for each butterfly wing based on reported microstructure data in the literature^19,21,58–64^. The colored letters in the figure correlate to the individual emissivity data in (c). The error bars represent the standard error for each data point, with the temperature data being taken from 3–8 different locations for each species. A linear fit to the data gives a correlation of *ε*_*mid-IR*_ = 0.0103*T*_*air*_ – 2.5867 (the dotted line represents the fit, with the gray band representing the 90% interval from the fit). The corresponding coefficient of correlation is + 0.86 and the coefficient of determination is + 0.74. (**c**) Computational emissivity predictions for the wing structures of various butterfly species from around the world. The plot depicts the emissivity in the mid-IR wavelengths, up to 14 µm. The optical property values were computed based on structural dimensions from annoying electron microscopy (SEM) images (sample size ranging from 10–100 unique wing locations) in existing literature^19,21,58–64^. Complete genus and species names are given in Fig. 4.

The computational models considered here have been used extensively in the optical physics literature^53–57^, and have also been previously used to analyze the experimentally determined mid-IR optical properties of butterfly wing microstructures^19,20^. The difference between measured emissivity and simulations does not exceed 0.1 in units of absolute emissivity, and our RCWA and FDTD simulations produce comparable results^19^, which validates their use in the current evaluation. Our studies make use of existing microstructure dimension data using methodology established in prior findings validated experimentally (Table S1, Figure S1)^19,21,58–64^ to newly evaluate the wing optical properties. The dimensions are used as input parameters for the RCWA computation, along with the refractive index and extinction coefficients of the materials involved (for instance, chitin^65–68^). We consider that the microstructures for all butterfly species are made of chitin, with the same refractive index values across the mid-IR wavelength range^65–68^. We find that in the mid-IR wavelengths, the butterfly wings attained emissivity values around 0.24–0.60 (Fig. 1). While butterflies are known to have regions of varying visible coloration across different regions of their wings, previous work has shown that regions of varying visible color have highly similar (or nearly identical) mid-IR emissivity values^19^. The likely reason for this is that the visible coloration and mid-IR emissivity arise from phenomena observed in two different wavelength ranges^16,19,20,44,47^. While visible coloration occurs in the 300–700 nm wavelength range, the mid-IR wavelength range extends from 7.5 µm to 14 µm wavelengths^16,19,20^. The interaction of light with photonic structures (such as the butterfly wing mesh) is largely scale-dependent; light of a given wavelength interacts strongly with structures of similar dimension scale. Consequently, the structural causes of coloration and mid-IR emissivity tend to be different in dimensional scale^19,20,44,47^. While structural coloration arises from diffraction and constructive interference caused by structures in the nanometer-scale, mid-IR optical properties are influenced by structures 10–100 times larger^19,47^. Diffraction and interference, primary causes of coloration and changes in emissivity, are thus highly wavelength selective responses of light interacting with photonic structures^19^. The varying values of emissivity observed in the mid-IR wavelengths could result in varying thermal performance of the butterfly wings under different environmental thermal conditions^31,32,69–71^. The findings indicate a possibility for radiative thermoregulation in butterfly wings by structural modulation of spectral emissivity.

### Correlation of spectral optical properties with environmental conditions

To examine the relationship between the optical properties of butterfly wings and the butterflies’ habitat climatic conditions we focus on the spectral average emissivity values in an understudied wavelength range of significance to thermoregulation. Specifically, the mid-IR spectrum (from 7.5 µm to 14 µm wavelengths, the atmospheric transmission window) is expected to offer radiative cooling of the butterflies by re-emission of heat from the butterfly wings to outer space^31,32^. We thus compared the average mid-IR emissivity of the butterfly wings to the annual average air temperature of the corresponding habitat range of the butterflies.

#### Correlation of mid-IR emissivity with annual average air temperature

We first used the Köppen-Geiger climate classifications^72,73^ to take into consideration the monthly average, maximum, and minimum values of air temperature and precipitation across all locations of the world (Fig. 1a). The use of monthly average, maximum, and minimum values reduces uncertainties that arise by simply considering annual average temperature due to the fluctuation in the values recorded temporally and spatially.

We then looked at the individual habitat air temperature data for each of the butterflies in more detail. In general, the air temperature varied with the latitude of measurement and the altitude of the location^73^. For instance, the northern plains in India experience a different air temperature (corresponding to a different climate classification) compared to the elevated regions of the Tibetan plateau, although both locations are along the same latitude. In order to avoid spatial variations in the air temperature data, the reported values were taken as the average of the reported data across at least 3 different weather stations within the geographic range of each of the butterflies^74,75^. The temporal variations in data were avoided by considering the annual average ambient air temperature for the analysis.

We then compared the mid-IR emissivity of the butterfly wings with the corresponding annual average air temperature values of their respective habitats (Fig. 1b). The mid-IR emissivity values are averaged for each butterfly within the wavelength range of 7.5–14 µm. We found a correlation between mid-IR emissivity and annual average air temperature, in which linear regression yielded a coefficient of correlation (*R*) of + 0.86. The corresponding coefficient of determination (*R*^2^) is + 0.74, indicating that 74% of the changes in the mid-IR emissivity can be explained by changes in the air temperature.

The mean value of the mid-IR emissivity for the entire dataset was 0.40, with a standard deviation of 0.13, with all values ranging between a minimum and maximum of 0.21 and 0.60 respectively (Fig. 1c). The butterfly *Celastrina echo* has an average mid-IR emissivity of 0.21, with an average habitat air temperature of less than 280 K (7 °C) annually (climate classification: Dfc)^72–75^. However, *Heliconius sara* has an average mid-IR emissivity of 0.60 and its annual average air temperature exceeds 290 K (17 °C) (climate classification: Af)^72–75^. We hypothesize that the mid-IR emissivity offers cooling by re-emission of heat from the butterfly wings across varying habitat climates^19,44^. Higher values of mid-IR emissivity result in greater heat loss from the wings due to re-emission of heat to outer space (at a temperature of 3 K or –270 °C). The results show a correlation between the butterflies’ habitat annual average ambient air temperature and the mid-IR emissivity of the butterfly wings.

We also analyzed the possible correlation between mid-IR emissivity and precipitation, another factor considered in the Köppen-Geiger climate classifications. The analysis showed wide variations in precipitation globally, with no evident direct link to either latitude or altitude^74,75^. Butterfly wings have previously been extensively studied for their hydrophobic characteristics^76–78^. However, thus far there is no established evidence for thermal adaptation to precipitation. We analyzed the annual average precipitation across the various habitats and plotted them on a relative scale from low to high (light blue to deep red) (Fig. 1b). Based on the analysis, there was no direct link between mid-IR emissivity and precipitation. While studies document the overall hydrophobic nature of butterfly wings, the dominant geometric parameters controlling hydrophobicity could differ from those controlling spectral emissivity^76–78^.

#### Effects of seasonal variations in air temperature

**Figure 2:**
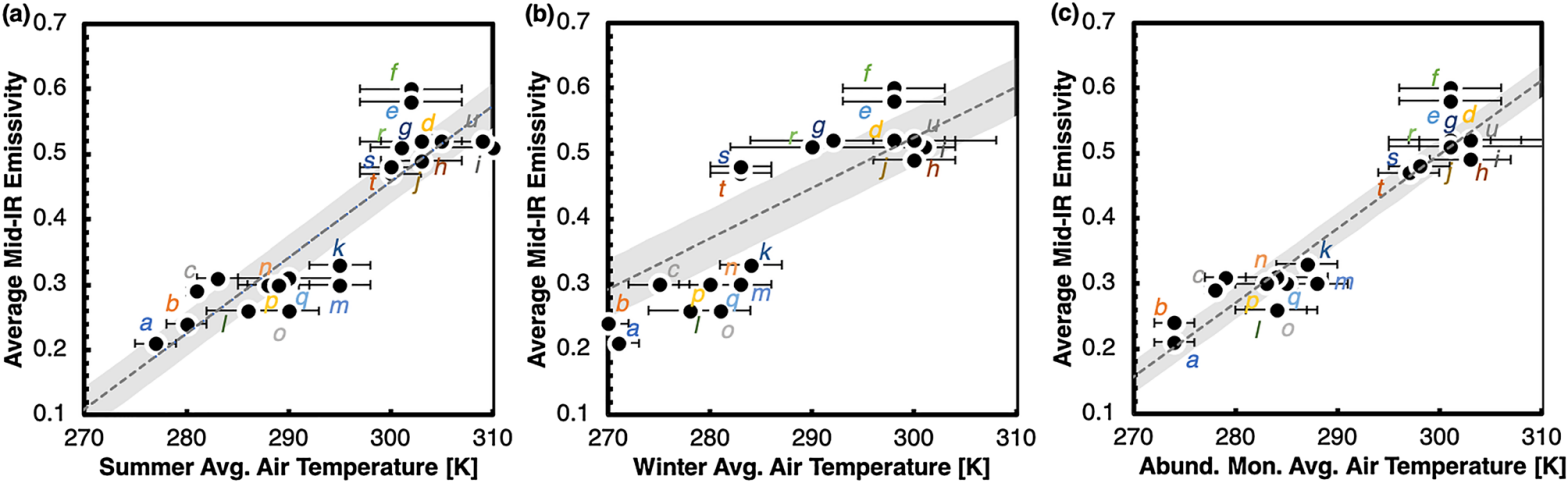
Comparison of the butterflies’ average wing mid-IR emissivity in relation to their habitat average air temperature during summer, winter, and most abundant months. (**a**) Average mid-IR emissivity of butterfly wings in relation to the summer average air temperature of their habitats (June–September in the northern hemisphere and December–March in the southern hemisphere). The linear correlation corresponds to *ε*_*mid-IR*_ = 0.0116*T*_*air*_ – 3.0354, with a coefficient of correlation of + 0.90. (**b**) Average mid-IR emissivity of butterfly wings in relation to the winter average air temperature of their habitats (December–March in the northern hemisphere and June–September in the southern hemisphere). The linear correlation corresponds to *ε*_*mid-IR*_ = 0.0077*T*_*air*_ – 1.7892, with a coefficient of correlation of + 0.82. (**c**) Average mid-IR emissivity of butterfly wings in relation to the average air temperature of their habitats during the butterflies’ most abundant months (Supplementary Table S1)^74,75^. The linear correlation corresponds to *ε*_*mid-IR*_ = 0.0114*T*_*air*_ – 2.9091, with a coefficient of correlation of + 0.95. In all the plots, the gray bands represent the 90% interval from the fit. The plots use the same methods of calculation and the same sources of information as Fig. 1. The error bars depict the standard error for each data point, with the temperature data ample from 3–8 different locations for each species. The colored letters in the figures correlate to the individual species' emissivity data in Fig. 1c.

In similar fashion, we compared the butterfly wing mid-IR emissivity with summer and winter average habitat air temperatures^74,75^. In addition to the seasonal analysis, we also analyzed the air temperatures of the months during which each of the butterflies are found in abundance in their habitats^19,21,58–64^. The results (Fig. 2) indicate correlations of the mid-IR emissivity of their wings to the summer (Fig. 2a), the winter (Fig. 2b), and abundant months’ (Fig. 2c) average air temperature. The coefficient of correlation for the summer data was + 0.90, the coefficient for the winter data was + 0.82, indicating that 81% and 67% of the variation in the mid-IR emissivity, respectively, can be explained by variation in summer and winter average air temperatures. It was interesting to observe that the coefficient of correlation was higher for summertime, during which many butterflies are active. The coefficient of correlation for the butterflies’ abundant months was even higher, at + 0.95, indicating that 90% of the changes in mid-IR emissivity can be explained by variation in air temperature. We deduce that there is a strong correlation between the abundant months’ habitat air temperature and the butterfly wing mid-IR optical properties.

Apart from seasonal variations, we also compare the effects of diurnal changes in air temperature (Figure S2). Though the results indicate noticeable correlations between daytime and nighttime air temperatures and mid-IR emissivity for the butterfly wings, it must be noted that butterflies are primarily active during daytime and spend the nights dormant. The nighttime air temperatures are also majorly influenced by daytime temperatures, and not much of significance may be deduced from such comparisons.

#### Correlation of mid-IR emissivity with other climatic factors

**Figure 3:**
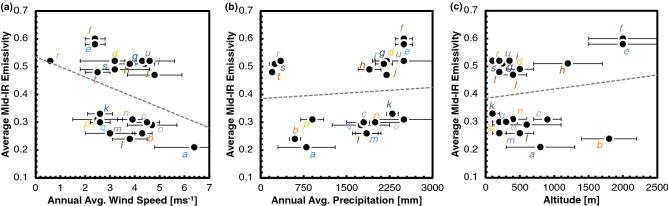
Comparison of average mid-IR emissivity of the butterfly wings to various climatic factors. (**a**) Average mid-IR emissivity of butterfly wings with respect to the annual average wind speed in their habitats^74,75^. The linear fit represents an equation of *ε*_*mid-IR*_ = −0.0381*wind speed* + 0.5376, with a coefficient of determination of 0.13 and a coefficient of correlation of -0.37. (**b**) Average mid-IR emissivity of butterfly wings with respect to the annual average precipitation in their habitats^74,75^. The linear fit represents an equation of *ε*_*mid-IR*_ = −1.0 × 10^−5^ *precipitation* + 0.3849, with a coefficient of determination of 0.01 and a coefficient of correlation of + 0.09. (**c**) Average mid-IR emissivity of butterfly wings with respect to the altitude in their habitats^74,75^. The linear fit represents an equation of *ε*_*mid-IR*_ = 3.0 × 10^–5^ *altitude* + 0.3852, with a coefficient of determination of 0.03 and a coefficient of correlation of + 0.16. The plots show a lack of correlation between habitat wind speed, precipitation, or altitude, and the optical properties of the butterfly wings. The plots use the same methods of calculation and the same sources of information as Fig. 1. The error bars depict the standard error for each data point, with the climatic data sampled from 3–8 different locations for each species. The colored letters in the figures correlate to the individual species' emissivity data in Fig. 1c.

In addition to analyzing the correlation between the optical properties of butterfly wings and the air temperature of their habitats, we also compared the mid-IR emissivity values for the butterfly wings with their respective habitat annual average wind speed, annual precipitation, and altitude (as altitude can be related to the air pressure) values^74,75^ (Fig. 3). The annual average wind speed values for the various habitats ranged between 2.4–6.4 ms^−1^. We observed (Fig. 3a) no significant correlation between the annual average wind speed and the mid-IR emissivity of the butterflies, with the coefficient of determination being 0.13 and a coefficient of correlation of -0.37. Similarly, the annual precipitation values ranged from 200–2500 mm. There was no noticeable correlation of the mid-IR emissivity to the annual precipitation (Fig. 3b), with a coefficient of determination of 0.01 and a coefficient of correlation of + 0.09. Finally, we considered the habitat altitude, which may be related to the air pressure. The butterflies inhabit locations ranging from 0–2000 m above sea level. Their wing mid-IR emissivity values do not show a correlation to the habitat altitude (Fig. 3c), with a coefficient of determination of 0.03 and a coefficient of correlation of 0.16. The lack of correlation confirms that wind speed, precipitation, and altitude do not play dominant roles in radiative heat transfer phenomena of butterfly wings in the process of re-emission of heat from their wings. We thus conclude that habitat air temperature extensively influences butterfly thermoregulation among the various climatic factors examined.

### Phylogenetic analysis

While a direct comparison of the mid-IR optical properties of butterfly wings to the butterflies' respective habitat air temperatures shows a positive correlation, it is important to examine whether or not this correlation still holds when the phylogenetic relatedness of the butterflies is taken into account. In order to ascertain that these traits evolved as adaptations to the butterflies’ habitat climatic conditions rather than as a result of phylogenetic inertia, or the tendency of related organisms to have related traits, we performed a phylogenetic independent contrasts analysis^79^. We utilized the methodology of Felsenstein’s contrasts^79–81^ by comparing the phylogeny-corrected mid-IR emissivity with the average annual air temperature.

First, we made use of a phylogenetic tree available from existing literature^82^. Several of the species whose optical properties we examined are present on the tree. However, where phylogenetic data were not available for a specific species, we substituted our studied species for closely related or sister species on that tree. The substitutions were made with species proximity taken into account—for example, sister species were substituted, failing which, substitute species were taken within the same tribe. The following substitutions on the tree were made for those species with no directly available phylogenetic data: *Papilio machaon* in place of *Papilio rumanzovia*, *Troides helena* in place of *Troides rhadamantus*, *Archaeoprepona demophoon* in place of *Prepona dexamenus*, *Heliconius sara and Heliconius doris* in place of *Heliconius melpomene*, *Euploeia mulciber* in place of * Danaus plexippus*, *Celastrina argiolus and Celastrina echo* in place of *Hemiargus ceraunus*, *Danis danis* in place of *Lepidochrysops patricia*, *Hypochrysops delicia* in place of *Lucia limbaria*, *Chrysozephyrus brillantinus* in place of *Artopoetes pryeri*, and *Arhopala japonica* in place of *Arhopala metamuta*.

**Figure 4:**
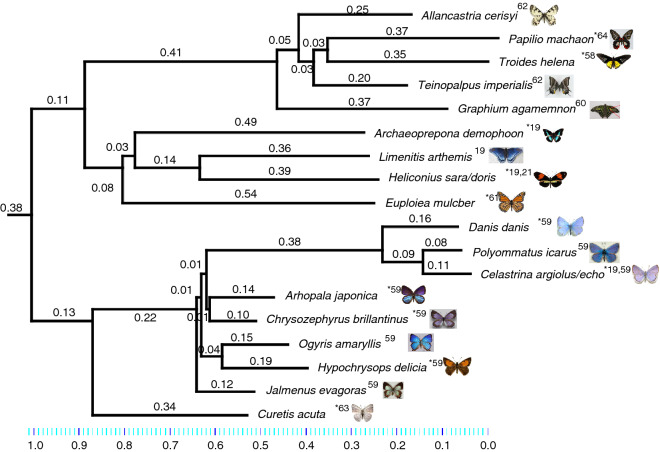
Phylogenetic tree used for the independent contrasts analysis for the species under consideration, based on an existing molecular phylogeny in the literature^82^. Where phylogenetic data were not directly available for the species in our study, closely related or sister species on the original phylogeny were substituted for Felsenstein’s contrasts analysis, as indicated in subsection 3 of our results. The numbers on the branches and the scale bar represent the branch length values in the original phylogeny. Stars indicate substituted species. References for species' SEM data are given as superscripts next to the species names.

A trimmed phylogenetic tree (Fig. 4) was then imported into Mesquite^83^ for independent contrasts analysis using the PDAP:PDTree package. For the species under consideration, we mapped their mid-IR emissivity and the annual average air temperature of their habitat on the trimmed tree. We then ran a Felsenstein’s contrasts analysis on the pair of characters, in order to deduce the phylogeny-corrected correlations between the mid-IR emissivity and air temperature. The use of the method was first validated by checking for the correlation (or lack of) between the contrasts of the characteristics and the standard deviation for the data (Figure S3). Once the use of the method was validated, we then analyzed the phylogeny-corrected correlations between the optical properties and the habitat climatic conditions. In order to account for *Heliconius melpomene* and *Hemiargus ceraunus* being substituted for 2 species each, we performed 4 sets of analyses for each possible permutation in order to obtain a range of possible results to account for the substitutions.

The results (Figure S4) depict a correlation between the contrasts for the mid-IR emissivity and annual average air temperature. For the correlation, Felsenstein’s contrasts correlation yielded an *R*^2^ value ranging between + 0.68 to + 0.71. The findings thus show that 68–71% of the variation in the mid-IR emissivity may be explained by variation in the air temperature. This result suggests that the optical properties of the butterflies’ wings evolved in response to the climatic conditions that they inhabit.

## Discussion

Our findings reveal one way in which butterflies from diverse geographic and climatic regions have thrived in their environment. Previous works have observed that butterflies make use of radiative heat transfer via their wing surfaces (especially in the mid-IR wavelengths), which aids in maintaining a wing temperature within a habitable temperature range^19,38,67^. However, while earlier studies considered individual butterfly species within the context of their own habitat climates or microclimates, our present study attempted to evaluate global trends in butterfly wing mid-IR optical properties in relation to climatic conditions worldwide. Our findings suggest that the mid-IR emissivity values of butterfly wings are correlated with the habitat air temperatures for the butterflies. That is to say, butterflies from regions with higher annual average air temperatures possess higher average mid-IR emissivity values than butterflies from regions with lower annual average air temperatures.

Our analyses also evaluated whether or not the microstructures responsible for the varying mid-IR emissivity values are the result of evolutionary processes leading to the adaptation of these butterflies to their habitat climatic conditions. A Felsenstein’s independent contrasts analysis verified the presence of a link between the mid-IR optical properties of the butterflies and the corresponding annual average air temperature in their habitats. By controlling for the effects of phylogenetic inertia on this correlation, we infer that wing microstructures and their consequent mid-IR emissivity values are at least in part the result of natural selection-driven adaptation to ambient air temperature.

Across the habitat climate conditions examined, butterfly wing mid-infrared emissivity appears to aid in butterfly thermoregulation by enhancing or inhibiting heat loss. We have attempted evaluations of solar spectrum reflectivity (Figures S5, S6) of the butterfly wings, and observe a weak correlation of around 16% with annual average solar irradiation (Fig. S7). Further studies of the microstructure-dependent optical and thermal properties of the butterflies such as the current one may facilitate the development of bio-inspired optical metamaterials and photonic structures that aid in radiative thermal management^31,70,84–86^.

Butterflies have adapted to the varying thermal demands put on them by making use of varying microstructures. Similarly, we may be able to overcome inherent material-dependent limitations on the optical properties and thermal behavior of various materials we work with by making use of surface microstructures to cater to varying optical and thermal needs. For example, the microstructure-dependent surface emissivity may be optimized for radiative heating or cooling by controlling the optical properties across various regions of the electromagnetic spectrum. An ideal radiative cooling surface, where the emissivity is selectively maximized in the mid-infrared wavelength region^32,69^ will advance current breakthroughs in thermal management across a wide array of applications—from space systems to wearable devices, and solar panels to building thermal management via optical coatings^87^. Similarly, an ideal radiative heating surface, where the emissivity is selectively maximized in the solar spectrum wavelengths will improve upon current solutions for just as wide an array of applications—ranging from thermophotovoltaics^88^ to personal thermal management via light-weight insulated heating fabrics for Arctic and Antarctic expeditions and space explorations^89^.

The results reported here could thus lay the ground for novel thermal management solutions and ultimately connect us closer to nature. Learning from the adaptations of butterflies to their habitat climates, we could invent and improve upon means for our own survival across extreme climates around the world.

## Conclusions

This work provides a new understanding of the relationship between the microstructure-dependent mid-infrared emissivity of butterfly wings to the butterflies’ habitat climates. The emissivity of butterfly wings in the mid-infrared wavelengths of 7.5–14 µm was computed using rigorous coupled-wave analysis and finite-difference time-domain methods based on existing microstructure microscopic images, a method previously validated experimentally^19^. The mid-infrared emissivity was then correlated to the butterflies’ habitat climate, via comparisons with the annual average air temperature, precipitation level, and wind speed. Apart from a comparison between mid-IR emissivity and the annual average air temperature, our analysis also examined the effects of diurnal and seasonal changes in climate. With butterflies being found in abundance in selective months of the year, and most being preferentially active during daytime, we observed that the correlation of these butterflies’ mid-IR emissivity values with the average air temperature for the time of year during which they are active was especially strong. We found a 90% correlation of the mid-infrared emissivity with the average air temperature for the months in which the butterflies are found in abundance.

In general, we noted that the mid-IR emissivity values of butterfly wings from warmer climates (with higher annual average air temperatures) are higher than those of butterflies from cooler climates (with lower annual average air temperature), which could be a habitat-dependent-thermoregulatory adaptation of the butterflies. To rule out the effects of phylogenetic inertia and to examine the role of evolution in the adaptations of these butterflies, we performed a Felsenstein’s independent contrasts analysis. The phylogeny-corrected data suggested that up to 71% of the variation in the mid-IR optical properties of the butterflies could be directly explained due to the corresponding annual average air temperature in their habitats.

Our findings suggest that butterfly wing mid-infrared emissivity plays a critical role in butterfly wing thermoregulation and that the ability of butterflies to adapt to climate change will be limited by the speed with which wing microstructures can be modified evolutionarily.

## Materials and methods

### Computation of optical properties

This work computes the spectral optical properties of butterfly wing microstructures using the rigorous couple wave analysis (RCWA) and finite-difference time-domain (FDTD)^57^ methods^32,49,52^. The RCWA method considers topographical variations and handles rigorous solutions of Maxwell’s equations^27–29^. Direct results from RCWA yield scattering matrices in the forward and reverse directions, from which reflectivity (*ρ*) and transmissivity (*τ*) are computed. The emissivity is assumed identical to the absorptivity by Kirchhoff’s law^27–29^ and is computed from the reflectivity and transmissivity (*α* = 1−*ρ*−*τ*). The FDTD method discretizes the samples and solves for space- and time-variant Maxwell’s equation for each unit cell. RCWA is semi-analytical and treats the waves and fields as sets of gratings, hence making it very effective for mesh-like structures. On the other hand, FDTD is superior in modeling curved surfaces and spherical shapes in full three-dimension. While FDTD is time-consuming, RCWA is more efficient towards optimizing the designs by variation of geometrical parameters. Here we used FDTD and RCWA as means for validating each other’s results.

In the work presented here, the butterfly wing microstructures are modeled as periodic arrays of mesh-like structures, whose structural parameters and dimensions are listed in Supplementary Section 1, Table S1. The spectral permittivity and complex refractive index of the chitin^65–67,91,92^ for the wings are input optical parameters for the spectral computations. Both the structural parameters (ridge periodicity (*a*), cross-link periodicity (*b*), ridge width (*c*), cross-link width (*d*), and height (*e*), detailed in Table S1 of the Supplementary Information) and optical parameters (spectral permittivity and complex refractive index of chitin) are inputted into custom built MATLAB (version 2020b) code in the case of RCWA, and commercially available Lumerical software (version 2020a) in the case of FDTD. The models are first described as mathematical arrays constituting the different materials that comprise the microstructures, and then the optical properties of the materials in each structural region are specified, which is comparable to building an outline first, followed by filling it in with material.

The simulation models are enclosed by boundaries that conform to the structural limits of the models themselves on four sides (along the X- and Y-axes)—the X–Y dimensions being defined by the ridge periodicity (*a*) and the cross-link periodicity (*b*). The remaining two sides, the top and bottom boundaries, extending for 10 times the model height (*e*), above and below (along the Z-axis) respectively. The boundary conditions used are as follows: perfectly matched layer boundary conditions at the top and bottom boundaries to effectively negate any erroneous reflection of light back into the model, and on the 4 sides of the model, periodic boundary conditions to emulate periodic repetitions of the mesh-like microstructures. The model was illuminated by a plane wave light source, with the wavelength ranging from 0.2 µm (200 nm) to 20 µm, although we primarily focused on the mid-IR wavelengths of 7.5–14 µm for our analyses in the work above. The models are discretized into meshes of 20 nm by 20 nm squares.

Once built, running the simulation yields two matrices as results, a forward propagation matrix and a backwards propagation matrix, respectively signifying the transmissivity and reflectivity of the structure. We then infer the emissivity as unity minus the sums of transmissivity and reflectivity^27,28^. The modeling code and corresponding optical properties can be accessed from the Materials and Correspondence section.

## Supplementary Information

Supplementary Information.

## References

[1] Kinoshita, S., *Structural Colors in the Realm of Nature* (World Scientific, 2008).

[2] Sun J, Bhushan B, Tong J (2013). Structural coloration in nature. RSC Adv..

[3] Whitney HM (2009). Floral iridescence, produced by diffractive optics, acts as a cue for animal pollinators. Science.

[4] Whitney HM, Kolle M, Alvarez-Fernandez R, Steiner U, Glover BJ (2009). Contributions of iridescence to floral patterning. Commun. Integr. Biol..

[5] Moyroud E (2017). Disorder in convergent floral nanostructures enhances signalling to bees. Nature.

[6] Mason CW (2005). Structural colors in feathers. II. J. Phys. Chem..

[7] Mason CW (2005). Structural colors in insects. III. J. Phys. Chem..

[8] Roberts NW, Marshall NJ, Cronin TW (2012). High levels of reflectivity and pointillist structural color in fish, cephalopods, and beetles. Proc. Natl. Acad. Sci..

[9] Zi J (2003). Coloration strategies in peacock feathers. Proc. Natl. Acad. Sci..

[10] McCoy DE, Feo T, Harvey TA, Prum RO (2018). Structural absorption by barbule microstructures of super black bird of paradise feathers. Nat. Commun..

[11] Teyssier J, Saenko SV, Van Der Marel D, Milinkovitch MC (2015). Photonic crystals cause active colour change in chameleons. Nat. Commun..

[12] Cooper KM, Hanlon RT, Budelmann BU (1990). Physiological color change in squid iridophores. Cell Tissue Res..

[13] Glover BJ, Whitney HM (2010). Structural colour and iridescence in plants: The poorly studied relations of pigment colour. Ann. Bot..

[14] Shi NN (2015). Keeping cool: Enhanced optical reflection and radiative heat dissipation in Saharan silver ants. Science.

[15] Preciado JA (2002). Radiative properties of polar bear hair. Am. Soc. Mech. Eng. Bioeng. Div..

[16] Bosi SG, Hayes J, Large MCJ, Poladian L (2008). Color, iridescence, and thermoregulation in Lepidoptera. Appl. Opt..

[17] Kinoshita S, Yoshioka S, Fujii Y, Okamoto N (2002). Photophysics of structural color in the *Morpho* butterflies. Forma-Tokyo.

[18] Tabata H, Kumazawa K, Funakawa M, Takimoto JI, Akimoto M (1996). Microstructures and optical properties of scales of butterfly wings. Opt. Rev..

[19] Krishna A (2020). Infrared optical and thermal properties of microstructures in butterfly wings. Proc. Natl. Acad. Sci. USA.

[20] Tsai CC (2020). Physical and behavioral adaptations to prevent overheating of the living wings of butterflies. Nat. Commun..

[21] Wilts BD, Vey AJM, Briscoe AD, Stavenga DG (2017). Longwing (*Heliconius*) butterflies combine a restricted set of pigmentary and structural coloration mechanisms. BMC Evol. Biol..

[22] Berthier S (2005). Thermoregulation and spectral selectivity of the tropical butterfly *Prepona meander*: A remarkable example of temperature auto-regulation. Appl. Phys. A Mater. Sci. Process..

[23] Vukusic P, Sambles JR (2003). Photonic structures in biology. Nature.

[24] Siddique RH, Diewald S, Leuthold J, Hölscher H (2013). Theoretical and experimental analysis of the structural pattern responsible for the iridescence of *Morpho* butterflies. Opt. Express.

[25] Steindorfer MA, Schmidt V, Belegratis M, Stadlober B, Krenn JR (2012). Detailed simulation of structural color generation inspired by the *Morpho* butterfly. Opt. Express.

[26] Munro JT (2019). Climate is a strong predictor of near-infrared reflectance but a poor predictor of colour in butterflies. Proc. R. Soc. B Biol. Sci..

[27] Incropera, F. P., DeWitt, D. P., Bergman, T. L. & Lavine, A. S. *Fundamentals of Heat and Mass Transfer* (Wiley, 2006).

[28] DeWitt, D. P., Incropera, F. P. “Physics of thermal radiation” in *Theory and Practice of Radiation Thermometry*, (1988), pp. 19–89.

[29] Howell, J. R., Menguc, M. P., Siegel, R. *Thermal Radiation Heat Transfer* (CRC Press, 2016).

[30] Lord, S. D. A new software tool for computing earth’s atmospheric transmission of near- and far-infrared radiation. *NASA Tech. Memo. 103957* (1992).

[31] Raman AP, Anoma MA, Zhu L, Rephaeli E, Fan S (2014). Passive radiative cooling below ambient air temperature under direct sunlight. Nature.

[32] Krishna A, Lee J (2018). Morphology-driven emissivity of microscale tree-like structures for radiative thermal management. Nanoscale Microscale Thermophys. Eng..

[33] Zhai Y (2017). Scalable-manufactured randomized glass-polymer hybrid metamaterial for daytime radiative cooling. Science.

[34] Zhang XA (2019). Dynamic gating of infrared radiation in a textile. Science.

[35] Xu C, Stiubianu GT, Gorodetsky AA (2018). Adaptive infrared-reflecting systems inspired by cephalopods. Science.

[36] Xie D (2019). Broadband omnidirectional light reflection and radiative heat dissipation in white beetles: *Goliathus goliatus*. Soft Matter.

[37] Heinrich B (1974). Thermoregulation in endothermic insects. Science.

[38] Kingsolver JG (1983). Thermoregulation and flight in *Colias* butterflies: elevational patterns and mechanistic limitations. Ecology.

[39] Rawlins JE (1980). Thermoregulation by the black swallowtail butterfly, *Papilio polyxenes* (Lepidoptera: Papilionidae). Ecology.

[40] Clench HK (1966). Behavioral thermoregulation in butterflies. Ecology.

[41] Bonebrake TC, Boggs CL, Stamberger JA, Deutsch CA, Ehrlich PR (2014). From global change to a butterfly flapping: Biophysics and behaviour affect tropical climate change impacts. Proc. R. Soc. B Biol. Sci..

[42] Nève G, Hall C (2016). Variation of thorax flight temperature among twenty Australian butterflies (Lepidoptera: Papilionidae, Nymphalidae, Pieridae, Hesperiidae, Lycaenidae). Eur. J. Entomol..

[43] MacLean HJ, Higgins JK, Buckley LB, Kingsolver JG (2016). Morphological and physiological determinants of local adaptation to climate in Rocky Mountain butterflies. Conserv. Physiol..

[44] Tsai, C. C., *et al.*, Butterflies regulate wing temperatures using radiative cooling in *2017 Conference on Lasers and Electro-Optics (CLEO)*, (IEEE, 2017), p. 9.

[45] Watanabe K, Hoshino T, Kanda K, Haruyama Y, Matsui S (2005). Brilliant blue observation from a *Morpho*-butterfly-scale quasi-structure. Jpn. J. Appl. Phys..

[46] Wilts BD, Giraldo MA, Stavenga DG (2016). Unique wing scale photonics of male Rajah Brooke’s birdwing butterflies. Front. Zool..

[47] De Keyser R, Breuker CJ, Hails RS, Dennis RLH, Shreeve TG (2015). Why small is beautiful: Wing colour is free from thermoregulatory constraint in the small lycaenid butterfly, Polyommatus icarus. Polyommatus icarus. PLoS One.

[48] Biró, L. P. *et al.*, Role of photonic-crystal-type structures in the thermal regulation of a lycaenid butterfly sister species pair. *Phys. Rev. E Stat. Physics, Plasmas, Fluids, Relat. Interdiscip. Top.* **67**, 7 (2003). 10.1103/PhysRevE.67.021907 12636715

[49] Sala-Casanovas M, Krishna A, Yu Z, Lee J (2019). Bio-inspired stretchable selective emitters based on corrugated nickel for personal thermal management. Nanoscale Microscale Thermophys. Eng..

[50] Phan L (2013). Reconfigurable infrared camouflage coatings from a cephalopod protein. Adv. Mater..

[51] Pris AD (2012). Towards high-speed imaging of infrared photons with bio-inspired nanoarchitectures. Nat. Photonics.

[52] Krishna A (2019). Ultraviolet to mid-infrared emissivity control by mechanically reconfigurable graphene. Nano Lett..

[53] Moharam MG, Gaylord TK (1981). Rigorous coupled-wave analysis of planar-grating diffraction. J. Opt. Soc. Am..

[54] Moharam, M. G. Coupled-wave analysis of two-dimensional dielectric gratings in *Holographic Optics: Design and Applications*, (1988), p. 8.

[55] Peng S, Morris GM (1995). Efficient implementation of rigorous coupled-wave analysis for surface-relief gratings. J. Opt. Soc. Am. A.

[56] Moharam MG, Gaylord TK, Grann EB, Pommet DA (1995). Formulation for stable and efficient implementation of the rigorous coupled-wave analysis of binary gratings. J. Opt. Soc. Am. A.

[57] Taflove, A., Hagness, S. C. *Computational Electrodynamics: The Finite-Difference Time-Domain Method* (Artech House, 2005).

[58] Fang J (2018). Enhanced photocatalytic hydrogen production on three-dimensional gold butterfly wing scales/CdS nanoparticles. Appl. Surf. Sci..

[59] Wilts BD, Leertouwer HL, Stavenga DG (2009). Imaging scatterometry and microspectrophotometry of lycaenid butterfly wing scales with perforated multilayers. J. R. Soc. Interface.

[60] Aideo, S. N., Mohanta, D. Investigation of manifestation of optical properties of butterfly wings with nanoscale zinc oxide incorporation. *J. Phys: Confer. Ser.* **765**, 012019 (2016).

[61] Guan Y (2018). Ordering of hollow Ag-Au nanospheres with butterfly wings as a biotemplate. Sci. Rep..

[62] Simonsen TJ (2011). Phylogenetics and divergence times of Papilioninae (Lepidoptera) with special reference to the enigmatic genera *Teinopalpus* and *Meandrusa*. Cladistics.

[63] Wilts BD, Pirih P, Arikawa K, Stavenga DG (2013). Shiny wing scales cause spec(tac)ular camouflage of the angled sunbeam butterfly, *Curetis acuta*. Biol. J. Linn. Soc..

[64] Wu L, Han Z, Qiu Z, Guan H, Ren L (2007). The microstructures of butterfly wing scales in northeast of China. J. Bionic Eng..

[65] Azofeifa DE, Arguedas HJ, Vargas WE (2012). Optical properties of chitin and chitosan biopolymers with application to structural color analysis. Opt. Mater. (Amst).

[66] Vargas WE, Azofeifa DE, Arguedas HJ (2013). Índices de refracción de la quitina, el quitosano y el ácido úrico con aplicación en análisis de color estructural. Opt. Pura y Apl..

[67] Herman A, Vandenbem C, Deparis O, Simonis P, Vigneron JP (2011). Nanoarchitecture in the black wings of Troides magellanus : A natural case of absorption enhancement in photonic materials. Nanophotonic Mater. VIII.

[68] Yoshioka S, Kinoshita S (2004). Wavelength-selective and anisotropic light-diffusing scale on the wing of the *Morpho* butterfly. Proc. Biol. Sci..

[69] Catalanotti S (1975). The radiative cooling of selective surfaces. Sol. Energy.

[70] Long Kou J, Jurado Z, Chen Z, Fan S, Minnich AJ (2017). Daytime radiative cooling using near-black infrared emitters. ACS Photonics.

[71] Wasserthal LT (1975). The role of butterfly wings in regulation of body temperature. J. Insect Physiol..

[72] Peel MC, Finlayson BL, McMahon TA (2007). Updated world map of the Köppen-Geiger climate classification. Hydrol. Earth Syst. Sci..

[73] New M, Lister D, Hulme M, Makin I (2002). A high-resolution data set of surface climate over global land areas. Clim. Res..

[74] Weather Spark Weather Data. https://weatherspark.com (July 10, 2019).

[75] Weather Underground Historical Weather. https://www.wunderground.com/history/ (August 2, 2018).

[76] Liu F (2011). Replication of homologous optical and hydrophobic features by templating wings of butterflies *Morpho menelaus*. Opt. Commun..

[77] Chen T, Cong Q, Qi Y, Jin J, Choy KL (2018). Hydrophobic durability characteristics of butterfly wing surface after freezing cycles towards the design of nature inspired anti-icing surfaces. PLoS ONE.

[78] Fang Y, Sun G, Wang TQ, Cong Q, Ren LQ (2007). Hydrophobicity mechanism of non-smooth pattern on surface of butterfly wing. Chin. Sci. Bull..

[79] Garland T, Harvey PH, Ives AR (1992). Procedures for the analysis of comparative data using phylogenetically independent contrasts. Syst. Biol..

[80] Felsenstein J (1985). Phylogenies and the comparative method. Am. Nat..

[81] Felsenstein J (1988). Phylogenies and quantitative characters. Annu. Rev. Ecol. Syst..

[82] Espeland M (2018). A comprehensive and dated phylogenomic analysis of butterflies. Curr. Biol..

[83] Maddison WP, Maddison DR (2008). Mesquite: a modular system for evolutionary analysis. 2010. Version.

[84] Cai, W., Shalaev, V. M. *Optical Metamaterials*, 10th Ed. (Springer, 2010).

[85] Zheludev NI, Kivshar YS (2012). From metamaterials to metadevices. Nat. Mater..

[86] Chen Z, Zhu L, Raman A, Fan S (2016). Radiative cooling to deep sub-freezing temperatures through a 24-h day-night cycle. Nat. Commun..

[87] Mandal J (2018). Hierarchically porous polymer coatings for highly efficient passive daytime radiative cooling. Science.

[88] Lenert A (2014). A nanophotonic solar thermophotovoltaic device. Nat. Nanotechnol..

[89] Quintiere J (1974). Radiative characteristics of fire fighters’ coat fabrics. Fire Technol..

[90] Energy Sector Management Assistance Program (ESMAP). Global Solar Atlas 2.1: Technical Report. https://globalsolaratlas.info (World Bank, December 2019).

[91] Yoshioka S, Kinoshita S (2011). Direct determination of the refractive index of natural multilayer systems. Phys. Rev. E.

[92] Leertouwer HL, Wilts BD, Stavenga DG (2011). Refractive index and dispersion of butterfly chitin and bird keratin measured by polarizing interference microscopy. Opt. Express.

