# Supplementary material for: Air temperature drives the evolution of mid-infrared optical properties of butterfly wings
Source: Sci Rep. 2021 Dec 17;11:24143. doi: 10.1038/s41598-021-02810-1 (PMC8683501; doi:10.1038/s41598-021-02810-1)
Supplement: Supplementary file 1 — Supplementary Information. [file 41598_2021_2810_MOESM1_ESM.doc]

**Air Temperature Drives the Evolution of Mid-Infrared Optical Properties of Butterfly Wings**

*Anirudh Krishna^1,2*^, Xiao Nie^2^, Adriana D. Briscoe^3^, Jaeho Lee^2^**

^1^ Intel Corporation, Hillsboro, OR 97124, United States of America

^2^ Department of Mechanical and Aerospace Engineering, University of California, Irvine, CA 92697, United States of America

^3^ Department of Ecology and Evolutionary Biology, University of California, Irvine, CA 92697, United States of America

*Corresponding authors: [anirudh.krishna@intel.com](mailto:anirudh.krishna@intel.com), [jaeholee@uci.edu](mailto:jaeholee@uci.edu)

# Supplementary Information

# Computation of optical properties of butterfly wing microstructures

We used scaled SEM images from existing literature (**Table S1**) (1–9) to extract the dimensions of the micromesh structures and used these measurements as input parameters for the RCWA computation, with material information such as refractive index taken for chitin (10–12). The microstructures consist of periodic ridges that are linked together by periodic transverse cross-links, which are located on top of a substrate. The findings for the microstructure data in literature was updated as of June 2020.

The microstructures for all species were taken as a mesh-like unit cell (**Figure S1**), and the simulation models were constructed using average geometric dimensions gathered from SEM images for each of the species. The dimension measurements were performed at 10-100 unique locations for each species, and are reported as average values in the table below. In cases were the transverse cross-links are irregular in structure in the SEM images of the butterfly wings (as in *Papilio machaon*, *Allancastria cerisyi*, and *Teinopalpus imperialis*), we measured the periodicity of these cross-links as the node-to-node distance of where the cross-links intersect with the ridges.

**Table S1.** Average geometric dimensions of the microstructures present in the butterfly wings in this study. The microstructures are predominantly in the form of a series of parallel ridges that are connected in the perpendicular direction by cross-links. The dimensional input parameters for the emissivity computation include the periodicity/pitch of the ridges (a) and the cross-links (b), and their respective thicknesses (c and d), and the chitin sub-layer thickness (e). The thickness (e) is taken assumed to be 1.2 µm, and does not appreciably affect computation results as explained in prior literature (1). The model schematic is depicted in **Figure S1** below. The average mid-IR emissivity as a result of the corresponding RCWA and FDTD computation is listed alongside each of the samples. Numbers in parentheses are references.

| **Sample Name** | **Abundance** | **Avg. Ann. Air Temperature [K]** (13, 14) | **a [µm]** | **b [µm]** | **c [µm]** | **d [µm]** | **e [µm]** | **Average Mid-IR Emissivity** | |
| --- | --- | --- | --- | --- | --- | --- | --- | --- | --- |
|  |  |  |  |  |  |  |  | **RCWA** | **FDTD** |
| *Allancastria cerisyi* (7) | March-July | 288 ± 3 | 1.2 | 0.7 | 0.5 | 0.1 | 1.2 | 0.48 | 0.39 |
| *Archaeoprepona demophoon* (1) | Year-round | 301 ± 3 | 2 | 0.5 | 0.5 | 0.2 | 1.2 | 0.51 | 0.44 |
| *Arhopala japonica* (4) | Year-round | 279 ± 2 | 2.5 | 1 | 0.2 | 0.1 | 1.2 | 0.31 | 0.24 |
| *Aricia icarioides* (4) | Year-round | 303 ± 8 | 1.5 | 1 | 0.4 | 0.1 | 1.2 | 0.52 | 0.43 |
| *Celastrina argiolus* (4) | April-July | 288 ± 3 | 2.1 | 0.9 | 0.1 | 0.1 | 1.2 | 0.26 | 0.26 |
| *Celastrina echo* (1) | April-July | 274 ± 2 | 2 | 1 | 0.25 | 0.2 | 1.2 | 0.21 | 0.19 |
| *Chrysozephyrus brillantinus* (4) | Year-round | 271 ± 1 | 2.5 | 1 | 0.1 | 0.1 | 1.2 | 0.29 | 0.24 |
| *Curetis acuta* (8) | April-October | 289 ± 3 | 2 | 1 | 0.2 | 0.1 | 1.2 | 0.30 | 0.28 |
| *Danis danis* (4) | Year-round | 289 ± 3 | 2 | 1.2 | 0.2 | 0.1 | 1.2 | 0.47 | 0.41 |
| *Euploea mulciber* (6) | April-October | 301 ± 4 | 1.2 | 0.8 | 0.1 | 0.1 | 1.2 | 0.49 | 0.43 |
| *Graphium agamemnon* (5) | May-September | 301 ± 4 | 1.2 | 0.7 | 0.2 | 0.1 | 1.2 | 0.52 | 0.44 |
| *Heliconius doris* | Year-round | 300 ± 5 | 1.2 | 0.6 | 0.5 | 0.2 | 1.2 | 0.58 | 0.48 |
| *Heliconius sara* (1,2) | Year-round | 300 ± 5 | 1 | 0.5 | 0.5 | 0.2 | 1.2 | 0.60 | 0.52 |
| *Hypochrysops delicia* (4) | Year-round | 303 ± 8 | 1.5 | 1 | 0.2 | 0.1 | 1.2 | 0.51 | 0.44 |
| *Jalmenus evagoras* (4) | Year-round | 288 ± 3 | 2 | 1 | 0.2 | 0.1 | 1.2 | 0.30 | 0.28 |
| *Limenitis arthemis* (1) | March-October | 287 ± 3 | 2 | 0.85 | 0.5 | 0.2 | 1.2 | 0.33 | 0.30 |
| *Ogyris amaryllis* (4) | Year-round | 288 ± 3 | 2.1 | 1.6 | 0.2 | 0.1 | 1.2 | 0.47 | 0.40 |
| *Papilio machaon* (9) | March-September | 281 ± 5 | 1.9 | 1 | 0.5 | 0.2 | 1.2 | 0.31 | 0.28 |
| *Polyommatus icarus* (4) | May-September | 284 ± 4 | 1.7 | 0.8 | 0.2 | 0.1 | 1.2 | 0.26 | 0.24 |
| *Teinopalpus imperialis* (7) | April-July | 284 ± 4 | 1.7 | 0.5 | 0.2 | 0.1 | 1.2 | 0.36 | 0.31 |
| *Troides helena* (3) | Year-round | 303 ± 8 | 1.2 | 0.5 | 0.2 | 0.1 | 1.2 | 0.52 | 0.43 |


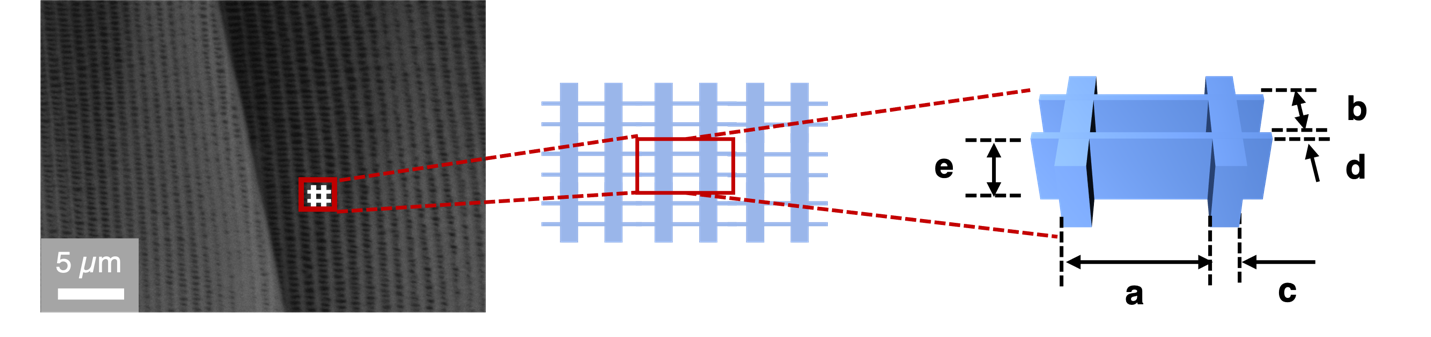


**Figure S1.** Schematic of model used for simulation of optical properties of butterfly wing microstructures. The SEM image depicts the wing microstructures present on the wings of *Heliconius doris*, from which we obtain the mesh-like unit cell. The various dimensions of the microstructure are listed using *a-e*, as detailed in **Table S1**.

# Effects of diurnal variations in air temperature

While a comparison of the mid-IR emissivity with the annual average air temperature showed a positive correlation, we still needed to examine the correlation between emissivity and habitat air temperature during daytime and during nighttime (15, 16). This is especially important since butterflies are more active during daylight hours, and relatively sedentary during night (17). We evaluated the correlation between the mid-IR emissivity of the butterfly wings and the daytime and nighttime annual average habitat air temperatures (**Figure S2**). The results indicated positive correlations of the mid-IR emissivity of butterfly wings to both their habitats’ daytime (**Figure S2a**) and nighttime (**Figure S2b**) annual average air temperature. The coefficient of correlation for the daytime data was +0.82, and for the nighttime data was +0.87, indicating that 68% and 76% of the variation in the butterflies’ mid-IR emissivity respectively can be explained by variation in the daytime and nighttime annual average air temperature of their habitats.


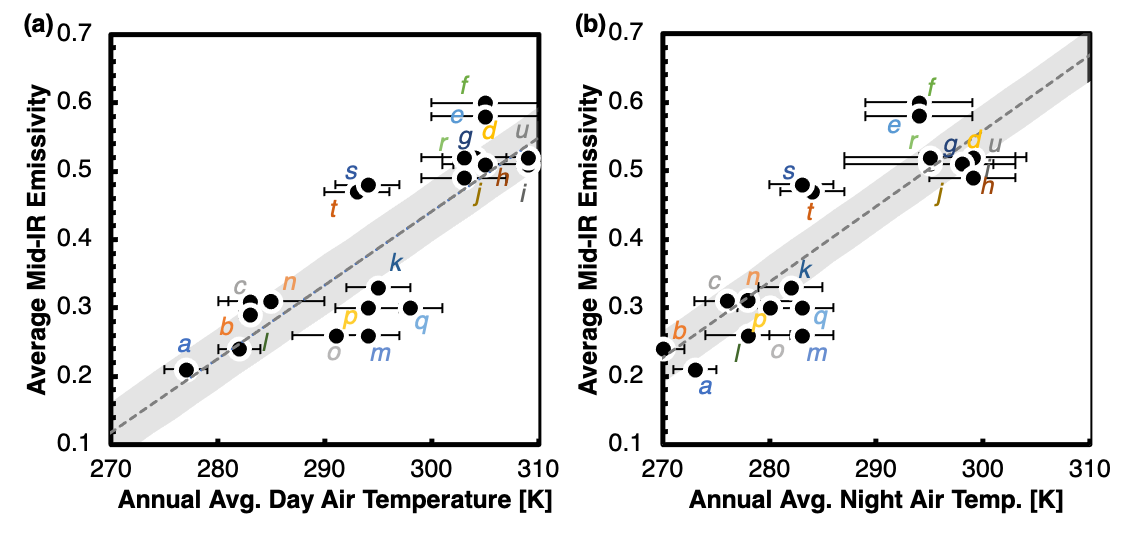


**Figure S2.** Comparison of butterfly wing mid-IR emissivity with daytime and nighttime annual average air temperature of the butterflies’ habitats (15, 16). (a) Butterfly wing average mid-IR emissivity in the wavelength range of 7.5–14 µm in relation to the daytime annual average air temperature of their habitats. The linear correlation corresponds to *ε_mid-IR_* = 0.0108*T_air_* – 2.7851, with a coefficient of correlation of +0.82. (b) Butterfly wing average mid-IR emissivity in the wavelength range of 7.5–14 µm in relation to the nighttime annual average air temperature of their habitats. The linear correlation corresponds to *ε_mid-IR_* = 0.011*T_air_* – 2.7549, with a coefficient of correlation of +0.87. The gray band in both plots corresponds to the 90% interval from the fit. The plots use the same method of calculations and the same sources of information as **Figure 1**. The error bars represent the standard error for each data point, with the temperature data being taken from 3-8 different locations for each species. The colored letters in the figures correlate to the individual emissivity data in **Figure 1c**.

# Phylogenetic independent contrasts analysis

In order to calculate the phylogeny-corrected correlations between the optical properties of butterfly wings and the butterflies’ habitat climatic conditions, we performed a phylogenetic independent contrasts analysis on the data (18). We first trimmed an existing phylogenetic tree (19) using the “prune clade” feature of Mesquite to include only the species under consideration, or closely related species. Where phylogenetic data were not available directly for species with computed emissivity values, we made the following substitutions: *Papilio machaon* in place of *Papilio rumanzovia* (both of the tribe Papilionini), *Troides helena* in place of *Troides rhadamantus* (both of the tribe Troidini), *Archaeoprepona demophoon* in place of *Prepona dexamenus* (both of the tribe Preponini), *Heliconius sara* and *Heliconius doris* in place of *Heliconius melpomene* (all of the tribe Heliconiini), *Euploeia mulciber* in place of *Danaus plexippus* (both of the tribe Danaini), *Celastrina argiolus* and *Celastrina echo* in place of *Hemiargus ceraunus* (all of the tribe Polyommatini), *Danis danis* in place of *Lepidochrysops patricia* (both of the tribe Polyommatini), *Hypochrysops delicia* in place of *Lucia limbaria* (both of the tribe Luciini), *Chrysozephyrus brillantinus* in place of *Artopoetes pryeri* (both of the tribe Theclini), and *Arhopala japonica* in place of *Arhopala metamuta* (both of the tribe Arhopalini). The resultant tree (**Figure 4**) was then used in the Mesquite (20) PDAP:PDTree software to deduce the phylogeny-corrected correlations.

In order to ascertain that the use of Felsenstein’s contrasts (21, 22) was valid for the data under consideration, we first needed to ensure that there exists no observable correlation between the absolute contrasts in each of the characters (optical properties and the habitat climatic conditions) with respect to the standard deviation in the data. We performed the analyses 4 times for each combination of possible species substitutions in the phylogenetic tree. The results (**Figure S3**) depict no such noticeable correlation for any of the characteristics, and hence validate the use of Felsenstein’s method for independent contrasts analysis in the present study.


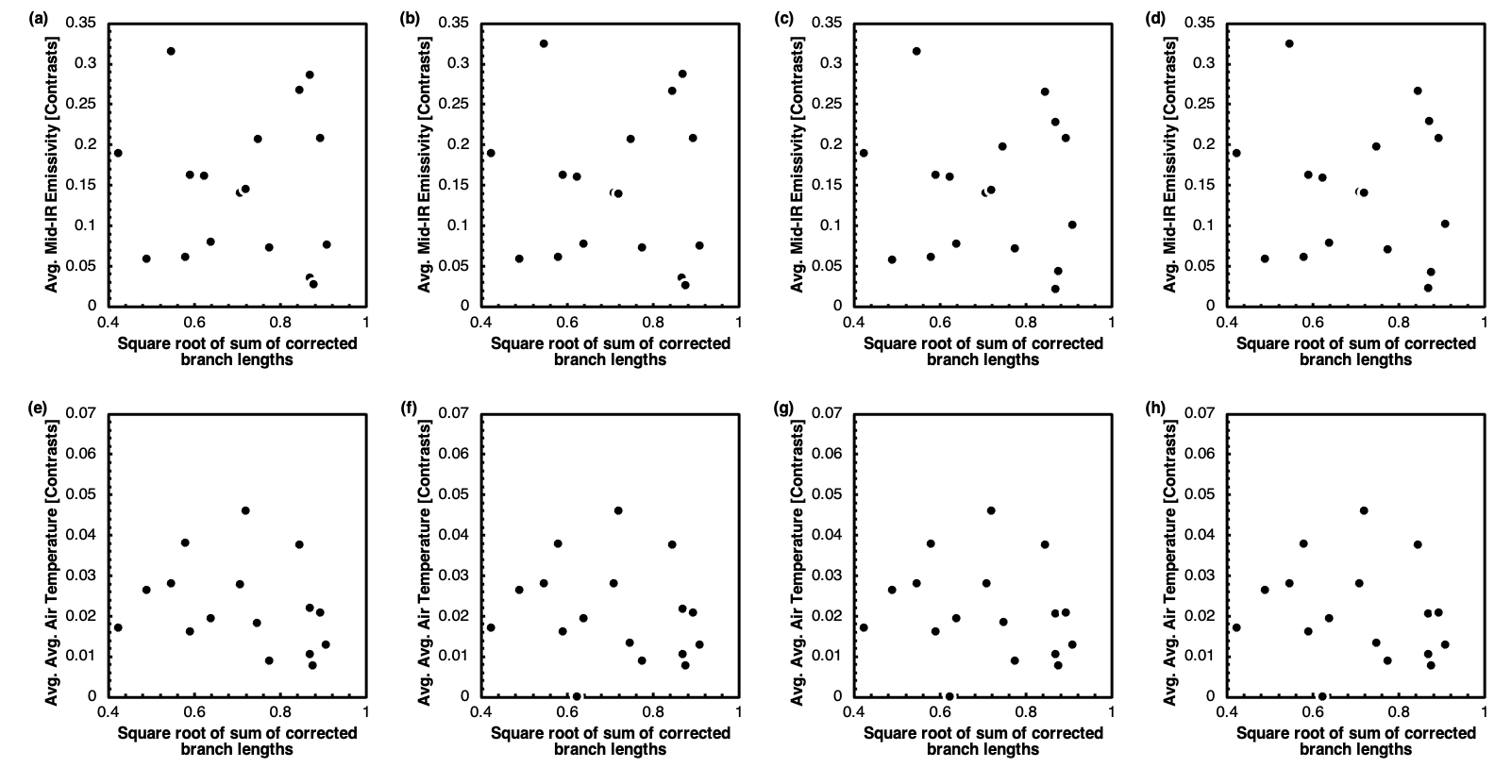


**Figure S3.** Comparison of average annual air temperature and average mid-IR emissivity used in the phylogenetic independent contrasts analysis with the standard deviation for the dataset. The characteristics used for the comparisons are (a-d) average annual air temperature for (a) *H. doris* in place of *H. melpomene* and *C. argiolus* in place of *H. ceraunus*, (b) *H. doris* in place of *H. melpomene* and *C. echo* in place of *H. ceraunus*, (c) *H. sara* in place of *H. melpomene* and *C. argiolus* in place of *H. ceraunus*, (d) *H. sara* in place of *H. melpomene* and *C. echo* in place of *H. ceraunus*, and (e-h) average mid-IR emissivity for (e) *H. doris* in place of *H. melpomene* and *C. argiolus* in place of *H. ceraunus*, (f) *H. doris* in place of *H. melpomene* and *C. echo* in place of *H. ceraunus*, (g) *H. sara* in place of *H. melpomene* and *C. argiolus* in place of *H. ceraunus*, (h) *H. sara* in place of *H. melpomene* and *C. echo* in place of *H. ceraunus*. The comparisons yield no observable correlation for either of the characteristics with respect to the standard deviation, validating the use of Felsenstein’s contrasts method.

We then deduced the phylogeny-corrected correlation between the mid-IR emissivity values and the annual average air temperature (**Figure S4**). The results from the PDAP:PDTree analysis depict an observable, linear correlation between the values, with the *R^2^* value for the correlation being between +0.68 and +0.71 for each possible combination of species substitutions in the phylogenetic tree.


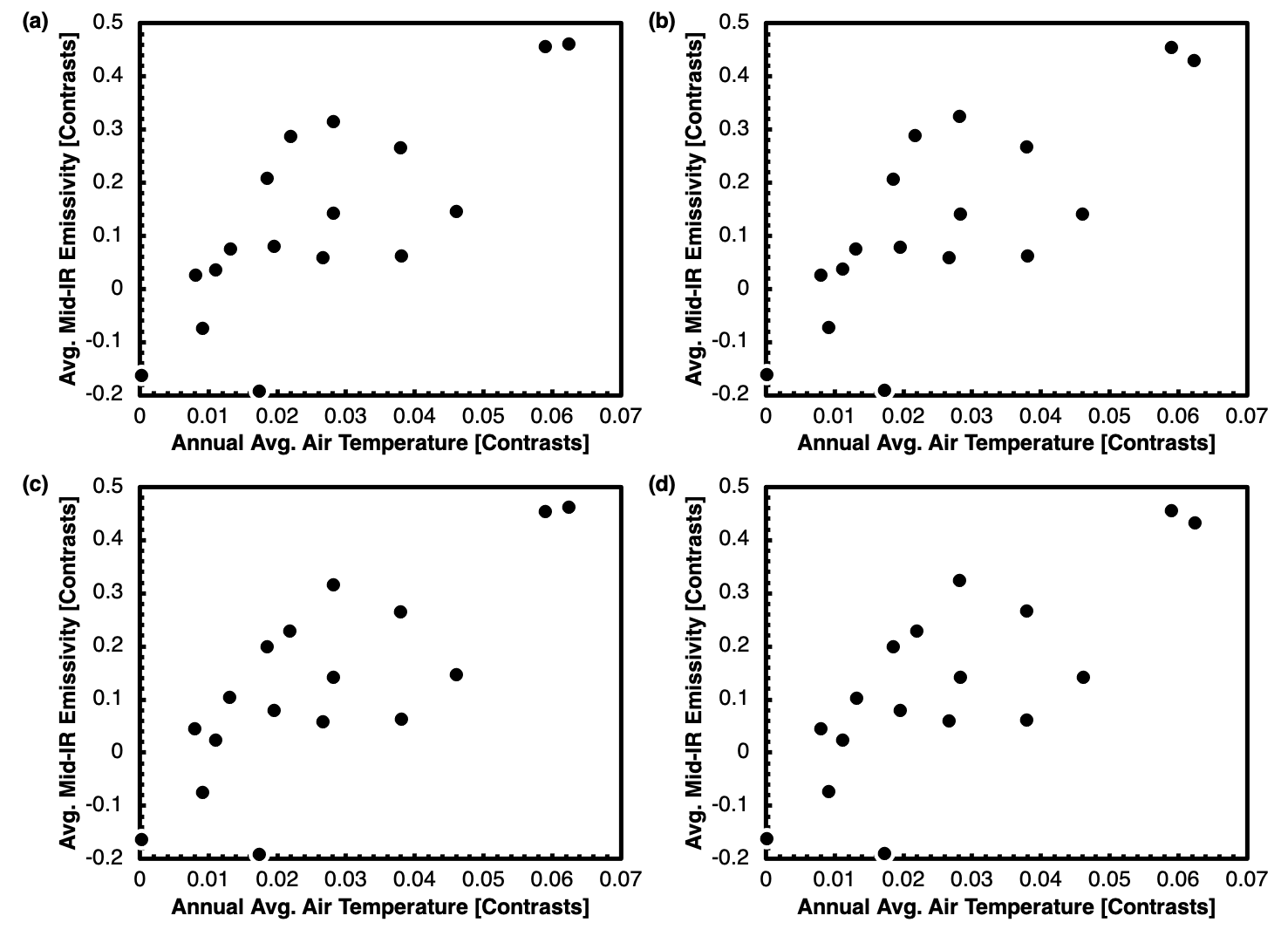


**Figure S4.** Felsenstein’s contrasts for the mid-IR emissivity against the annual average air temperature for (a) *H. doris* in place of *H. melpomene* and *C. argiolus* in place of *H. ceraunus*, (b) *H. doris* in place of *H. melpomene* and *C. echo* in place of *H. ceraunus*, (c) *H. sara* in place of *H. melpomene* and *C. argiolus* in place of *H. ceraunus*, (d) *H. sara* in place of *H. melpomene* and *C. echo* in place of *H. ceraunus*. The results depict a positive correlation, with the *R^2^* value being between +0.68 and +0.71, signifying that 68-71% of the changes in the average mid-IR emissivity can be explained by variation in the average annual air temperature for the butterflies’ habitat.

# Comparison of solar spectrum optical properties

We used SEM information from existing literature (1–9) as input parameters for the computation, with material information such as refractive index taken for chitin (10–12). The results (**Figure S5**) depict a variation of solar spectrum reflectivity values across the various butterfly species, which was then used for correlation analysis with their respective habitat climates.


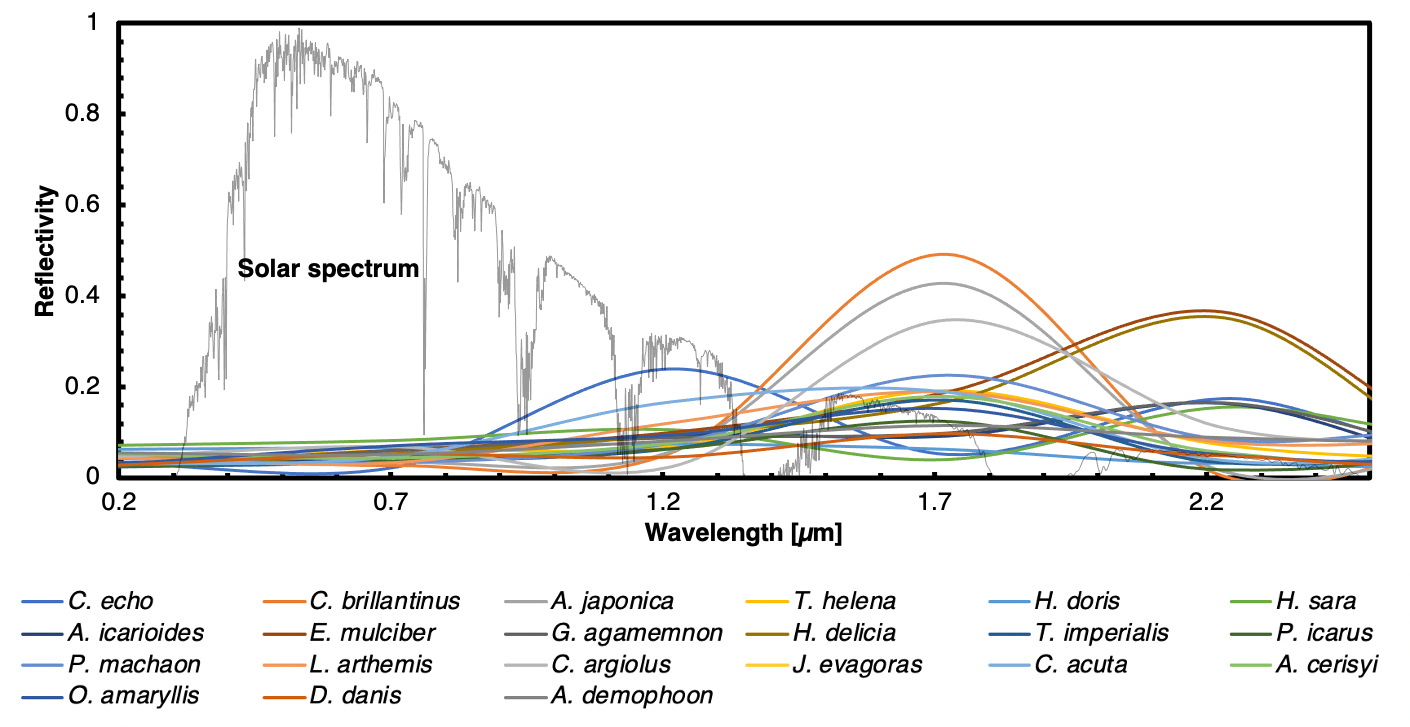


**Figure S5.** Computational reflectivity predictions for the wing structures of various butterfly species from around the world, depicting the reflectivity in the solar spectrum from 0.2 µm to 2.5 µm. The optical property values are computed based on structural dimensions from SEM analyses in existing literature (1–9).

#### **Correlation of solar reflectivity with annual average air temperature**

In a similar manner to the analysis of the mid-IR emissivity, we proceeded to compare the average solar reflectivity values for the butterfly wings with the annual average air temperature for their respective habitats. Solar reflectivity is a measure of the effectiveness of a material in reflecting incident solar energy, and is given as the ratio of the reflected radiance to the incident solar radiance, while emissivity is a measure of the effectiveness of a material in emitting radiant heat. The average solar reflectivity is taken for the wavelength range of 0.2–2.5 µm, corresponding to the incoming incident solar spectrum (23). The average annual air temperature values were taken for the habitats of the butterflies from existing literature (**Figure S6a**) (13–16). The results for the comparison depicted no direct correlation between the solar reflectivity and the air temperature (**Figure S6b**). For example, *Celastrina echo* depicted an average solar reflectivity of 0.10 with an annual average air temperature of 274±2 K (1±2˚C), while *Troides helena* showed an average solar reflectivity of 0.12 but with an annual average air temperature of 303±8 K (30±8˚C).


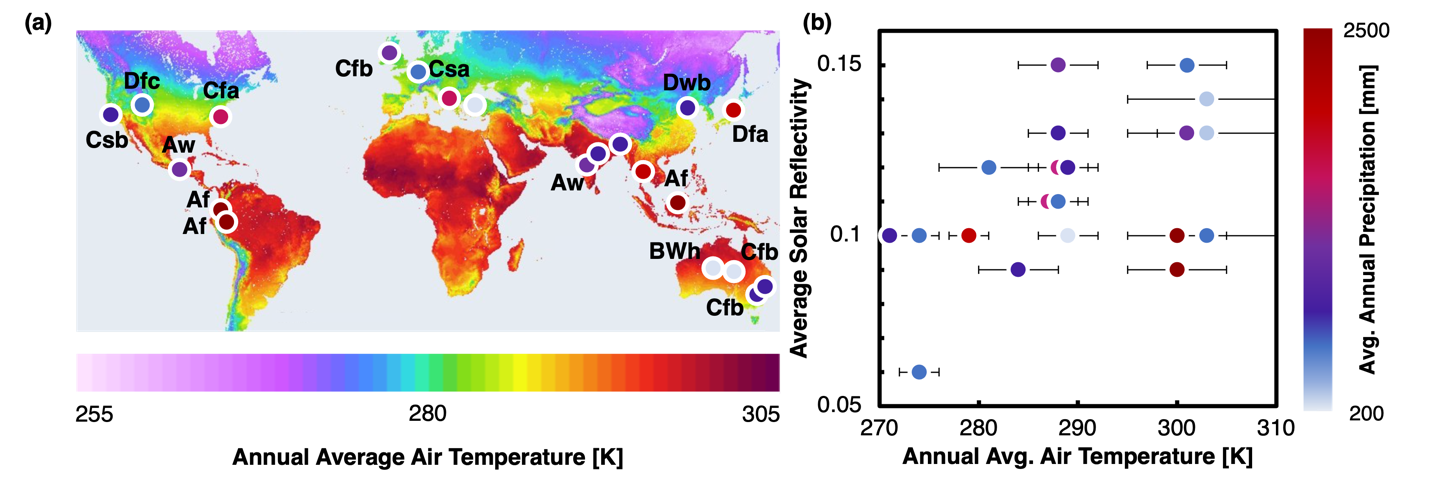


**Figure S6.** (a) Geographical mapping of the sampled butterfly species around the world, with annual average air temperature (14–16), generated using open source QGIS software v.3.20.3 ([www.qgis.org](http://www.qgis.org/)). (b) Average reflectivity in the wavelength range of 0.2–2.5 μm or the solar reflectivity of butterfly wings with respect to the annual average air temperature in their habitats. The results depict no noticeable correlation between the solar reflectivity and the annual average air temperature values for the respective butterflies’ habitats.

#### **Correlation of solar reflectivity with annual average solar irradiation**

Because solar reflectivity is the fraction of incident solar irradiation reflected by the butterfly wings, we proceeded to test for any possible correlation between annual average solar irradiation and average solar spectrum reflectivity, to check if butterfly wings are more reflective in habitats with higher incident sunlight. We thus chose the incoming annual average solar irradiation (23, 24) as an arbiter for our solar reflectivity data analysis. The annual average solar irradiation values do not solely possess a direct correlation to the latitude of measurement, and can vary depending on various factors such as forest cover and altitude (13, 25). For instance, while Florida, USA, and the Sahara Desert remain within comparable latitudes, their annual average solar irradiation values are vastly different (**Figure S7a**). Similarly, the eastern slopes of the Himalayas and coastal Brazil experience similar annual average solar irradiation values while being from vastly different altitudes (**Figure S7a**). Our choice of annual average solar irradiation was guided by the understanding that high solar reflectivity would result in low absorption of incoming solar irradiation, and then consequently result in lower heating capabilities for the butterfly wings.

The analysis showed a positive correlation between the average solar reflectivity (0.2–2.5 µm wavelengths) for the wings with the annual average solar irradiation for the respective habitats (**Figure S7b**). We noticed an increase in the solar reflectivity in butterflies from regions with higher annual average solar irradiation. Our understanding of the results suggests a potential correlation to the locational annual average solar irradiation and the butterflies’ solar reflectivity as an adaptation for control over the amount of heat absorbed by the butterfly wings from the incident sunlight.

The mean solar reflectivity for the entire dataset was 0.12, with a standard deviation of 0.04. The solar reflectivity values ranged between a minimum of 0.06 to a maximum of 0.15. The analysis of correlating the solar reflectivity with the annual average solar irradiation yielded a coefficient of correlation of +0.4 and a coefficient of determination of +0.16.


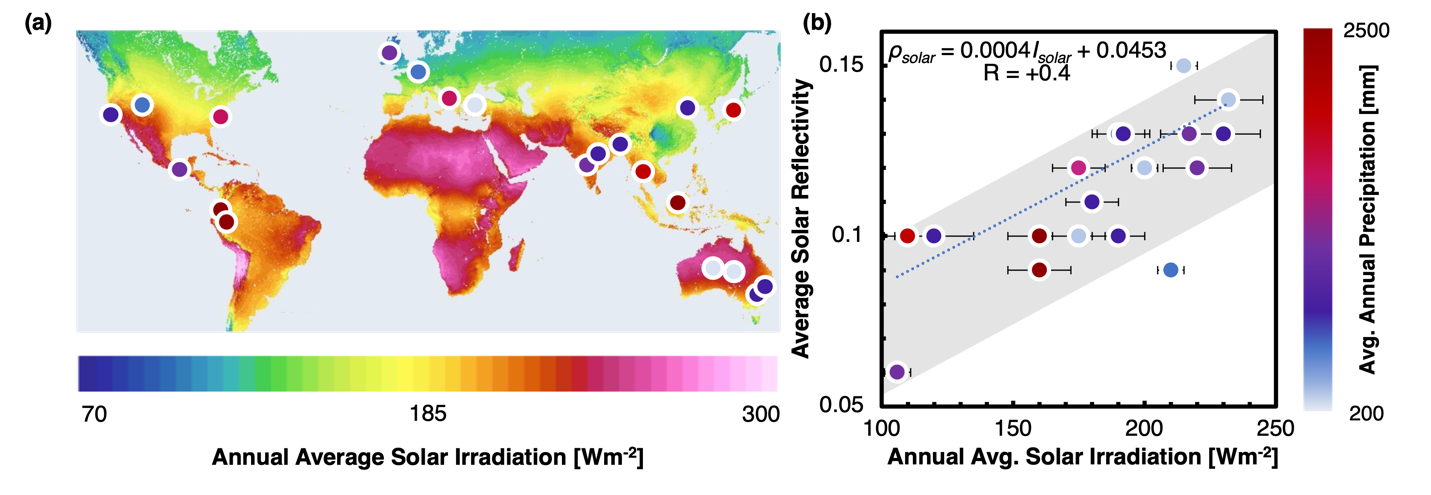


**Figure S7.** (a) Geographical mapping of the sampled butterfly species around the world, with annual average solar irradiation (23, 24), generated using open source QGIS software v.3.20.3 ([www.qgis.org](http://www.qgis.org/)). (b) Average reflectivity in the wavelength range of 0.2-2.5 μm or the solar reflectivity of butterfly wings with respect to the annual average solar irradiation in their habitats (23, 24). For a linear fit to the presented data set, the coefficient of correlation is +0.40 and the coefficient of determination is +0.16.

**References**

1. A. Krishna, *et al.*, Infrared optical and thermal properties of microstructures in butterfly wings. *Proc. Natl. Acad. Sci. U. S. A.* **117**, 1566–1572 (2020).

2. B. D. Wilts, A. J. M. Vey, A. D. Briscoe, D. G. Stavenga, Longwing (*Heliconius*) butterflies combine a restricted set of pigmentary and structural coloration mechanisms. *BMC Evol. Biol.* **17**, 1–12 (2017).

3. J. Fang, *et al.*, Enhanced photocatalytic hydrogen production on three-dimensional gold butterfly wing scales/CdS nanoparticles. *Appl. Surf. Sci.* **427**, 807–812 (2018).

4. B. D. Wilts, H. L. Leertouwer, D. G. Stavenga, Imaging scatterometry and microspectrophotometry of lycaenid butterfly wing scales with perforated multilayers. *J. R. Soc. Interface* **6**, S185–S192 (2009).

5. S. N. Aideo, D. Mohanta, Investigation of manifestation of optical properties of butterfly wings with nanoscale zinc oxide incorporation in *J. Physics: Conf. Ser.*, (2016).

6. Y. Guan, *et al.*, Ordering of hollow Ag-Au nanospheres with butterfly wings as a biotemplate. *Sci. Rep.* **8**, 1–7 (2018).

7. T. J. Simonsen, *et al.*, Phylogenetics and divergence times of Papilioninae (Lepidoptera) with special reference to the enigmatic genera *Teinopalpus* and *Meandrusa*. *Cladistics* **27**, 113–137 (2011).

8. B. D. Wilts, P. Pirih, K. Arikawa, D. G. Stavenga, Shiny wing scales cause spec(tac)ular camouflage of the angled sunbeam butterfly, *Curetis* *acuta*. *Biol. J. Linn. Soc.* **109**, 279–289 (2013).

9. L. Wu, Z. Han, Z. Qiu, H. Guan, L. Ren, The microstructures of butterfly wing scales in northeast of China. *J. Bionic Eng.* **4**, 47–52 (2007).

10. D. E. Azofeifa, H. J. Arguedas, W. E. Vargas, Optical properties of chitin and chitosan biopolymers with application to structural color analysis. *Opt. Mater. (Amst).* **35**, 175–183 (2012).

11. W. E. Vargas, D. E. Azofeifa, H. J. Arguedas, Índices de refracción de la quitina, el quitosano y el ácido úrico con aplicación en análisis de color estructural. *Opt. Pura y Apl.* **46**, 55–72 (2013).

12. A. Herman, C. Vandenbem, O. Deparis, P. Simonis, J. P. Vigneron, Nanoarchitecture in the black wings of *Troides* *magellanus*: a natural case of absorption enhancement in photonic materials. *Nanophotonic Mater. VIII* **8094**, 80940H (2011).

13. M. New, D. Lister, M. Hulme, I. Makin, A high-resolution data set of surface climate over global land areas. *Clim. Res.* **21**, 1–25 (2002).

14. M. C. Peel, B. L. Finlayson, T. A. McMahon, Updated world map of the Köppen-Geiger climate classification. *Hydrol. Earth Syst. Sci.* **11**, 1633–1644 (2007).

15. Weather Spark Weather Data. *https://weatherspark.com* (July 10, 2019).

16. Weather Underground Historical Weather. *https://www.wunderground.com/history/* (August 2, 2018).

17. H. K. Clench, Behavioral Thermoregulation in Butterflies. *Ecology* **47**, 1021–1034 (1966).

18. T. Garland, P. H. Harvey, A. R. Ives, Procedures for the analysis of comparative data using phylogenetically independent contrasts. *Syst. Biol.* **41**, 18–32 (1992).

19. M. Espeland, *et al.*, A comprehensive and dated phylogenomic analysis of butterflies. *Curr. Biol.* **28**, 770-778.e5 (2018).

20. W. P. Maddison, D. R. Maddison, Mesquite: a modular system for evolutionary analysis. 2010. *Version* **2**, 73 (2008).

21. J. Felsenstein, Phylogenies and the comparative method. *Am. Nat.* **125**, 1–15 (1985).

22. J. Felsenstein, Phylogenies and quantitative characters. *Annu. Rev. Ecol. Syst.* **19**, 445–471 (1988).

23. The National Renewable Energy Laboratory (NREL), Reference Solar Spectral Irradiance: Air Mass 1.5. *Am. Soc. Test. Mater. - Terr. Ref. Spectra Photovolt. Perform. Eval.*, 21 (2012).

24. Energy Sector Management Assistance Program (ESMAP). Global Solar Atlas 2.1: Technical Report. *https://globalsolaratlas.info* (World Bank, December 2019).

25. O. Coddington, J. L. Lean, P. Pilewskie, M. Snow, D. Lindholm, A solar irradiance climate data record. *Bull. Am. Meteorol. Soc.* **97**, 1265–1282 (2016).
